# Prevalence and Predictive Factors for Leg Ulcers in Sickle Cell Disease Patients in Saudi Arabia: A Cross-Sectional Observational Study

**DOI:** 10.7759/cureus.11280

**Published:** 2020-10-31

**Authors:** Feroze Kaliyadan, Ahmed Z Alkhars, Alreem A Albaqshi, Hajar M AlHajri, Norah K Albaqshi, Rawan M Aldihnayn, Zainab Y Almarzooq

**Affiliations:** 1 Dermatology, College of Medicine, King Faisal University, Al-Ahsa, SAU; 2 General Medicine and Surgery, College of Medicine, King Faisal University, Al-Ahsa, SAU; 3 Medicine, College of Medicine, King Faisal University, Al-Ahsa, SAU

**Keywords:** sickle cell disease, prevalence, leg ulcers, saudi arabia

## Abstract

Introduction

Saudi Arabia has a high prevalence of sickle cell disease (SCD). Leg ulceration is one of the complications associated with SCD. There is a gap in the literature in regard to the prevalence of leg ulcers among SCD patients in Saudi Arabia.

Objectives

The primary objective of this study was to evaluate the lifetime prevalence of leg ulcers in SCD patients in our population and to study the predictive factors of leg ulcers by using sociodemographic factors, clinical manifestations of SCD, and other relevant factors like hydroxyurea.

Methods

A cross-sectional study design was utilized. Data collection was done using an electronic survey to collect self-reported information for the prevalence of leg ulcers and possible associated factors. The survey was distributed using social media platforms. Chi-square test was used to test for the presence of an association between having leg ulcers and sociodemographic variables as well as SCD related history. Furthermore, binary logistic regression was utilized to determine factors predicting the incidence of leg ulcers among SCD.

Results

A total of 790 valid responses were included in the study. Among these, 646 were included in the analysis of leg ulcers prevalence. From them, 52 (8%) SCD patients reported a history of leg ulcers. The male to female ratio was (9.7% vs 7.2%). The age group most affected by leg ulcers was those older than 50 (16.7%). There was no significant association between a history of leg ulcers and sociodemographic variables. The only predictive factors for leg ulcers were having six to eight vaso-occlusive crises per month and having more than eight vaso-occlusive crises per month.

Conclusion

Leg ulcers among SCD patients in Saudi Arabia were considerably prevalent (8%). There was no statistically significant correlation between leg ulceration and sociodemographic variables. Leg ulcers were more likely in patients with a history of highly frequent vaso-occlusive crises. No association was found between the incidence of leg ulcers and other complications of sickle cell disease or hydroxyurea.

## Introduction

Sickle cell disease (SCD) is an autosomal recessive hemoglobinopathy characterized by the formation of abnormal red blood cell (RBC) shape with defective hemoglobin called hemoglobin S (HbS) [1]. The genotype (HbS) is caused by a mutation in the sixth amino acid of the beta-globin gene substituting a hydrophobic valine amino acid for glutamic acid. This change makes the HbS molecules more likely to polymerize in states of dehydration, hypoxia, or acidosis, affecting the RBCs shape to change from a normal biconcave disc shape to a sickle shape [2]. The change to a sickle shape results in anemia, and vaso-occlusion which in turn leads to SCD-associated clinical manifestations [3-5]. With time SCD can cause complications such as acute chest syndrome, priapism, pulmonary hypertension, cerebrovascular diseases, retinopathy, and skin ulcers [5].

Skin ulcers represent one of the most common and the most distressing cutaneous complication of SCD with a reported incidence that reach up to 25%-75% [6,7]. Chronic and recurrent ulcers can significantly affect the quality of life of sickle cell patients. Ulceration occurs due to poor circulation and perfusion especially in areas that have thin skin and low subcutaneous fat, like the medial and lateral malleoli, anterior tibia, dorsum, and the area overlying the Achilles tendon [2]. The pathogenesis of leg ulcers in SCD is not fully understood. However, there are several complex interactions of factors that are thought to play a role in the pathophysiology. For example, trauma can cause sickle cell ulcers by stimulating the sickling of RBCs, which leads to obstruction of the vessels, ending up with ischemia and tissue necrosis. Also, the elevation of venous blood pressure resulting from venous insufficiency could contribute to the formation of sickle cell ulcers. Moreover, anemia may play a major role in hypoxia causing oxygen deficiency and tissue necrosis. As well as for thrombocytosis when it presents in a patient with SCD, it leads to increased blood viscosity, promoting thrombosis and vascular blockage [8].

SCD is the most common single-gene blood disorder worldwide accounting for 70% of total hemoglobinopathies [7]. Studies have reported that SCD is highly prevalent in Saudi Arabia with an estimation of 0.5% for sickle cell trait (SCT) and 0.038% for SCD through the screening done by the premarital program. Newborn screening estimated a prevalence of 21% for SCT and 2.6% for SCD [9].

Although SCD is endemic in Saudi Arabia and despite the recognition of leg ulcers as a commonly encountered complication in sickle cell patients, to the best of our knowledge, no previous studies have screened the prevalence of leg ulcers among sickle cell patients in Saudi Arabia. As there are no existing studies, it would be important to address this gap in the literature. In this study, we aim to determine the lifetime prevalence of leg ulcers in sickle cell patients in Saudi Arabia and its predictive factors.

## Materials and methods

Study design and population

 A cross-sectional study targeting sickle cell patients living in Saudi Arabia to screen for the prevalence of leg ulcers among them. The study was conducted between August 2020 and October 2020.

Study tool and its validation

 A survey was constructed and developed by the investigators and was presented to specialists in dermatology and medicine who revised it, improved it, and then approved it. The questionnaire was constructed in English and then translated to Arabic for it to be comprehensible for the targeted population. The Arabic version was first examined by three different language experts and the translation was approved after grammatic and linguistic modifications. After that, a pilot study was performed on a small group of people (15 persons) to confirm a uniform understanding of the questions.

Data collection

An online survey was created using Google forms for data collection. The online survey was disseminated to the community using social media platforms such as Twitter, Instagram, and WhatsApp. Volunteers were recruited for data collection from areas known to have a high prevalence of SCD in Saudi Arabia. Eight persons were recruited from the Eastern region, two from the Western region, five persons from the Southern region, and four persons from the Northern region. Although the questionnaire was disseminated to the general population, only sickle cell patients were invited to participate as they were the target. Data were obtained through an online self-administered questionnaire where participants first consent to participate in the study before starting to fill the questionnaire. The questionnaire included two sections, the first section has only one question which is if they had sickle cell disease or not. Only those who answered they had sickle cell disease were able to continue to the second section, those answering they were carrier and those answering they were free from the disease and trait were not allowed to proceed further. The second section contained four parts. The first part surveyed sociodemographic data including age, gender, nationality, level of education, and marital status. The second part asked about SCD related history including frequency of admission per year, frequency of vaso-occlusive crises per month, previous incidence of SCD complications (acute chest syndrome, priapism, and stroke), and usage of Hydroxyurea. The third part screened for the presence of other causes of leg ulcers including thalassemia, chronic venous insufficiency, and systemic lupus erythematosus. The fourth and the last part was about the dermatological aspects of SCD which surveyed the prevalence of leg ulcers, their frequency, were they painful or not and if they were associated with vaso-occlusive crises or not, the investigation that was conducted to be diagnosed with leg ulcers, the treatment plan for leg ulcers, and the duration of healing time.

Sampling and sample size

The used sampling technique was convenient random sampling where an online questionnaire was dispersed on social media platforms and sickle cell patients were invited to fill it up. The sample size was calculated using the formula n = z2pq\d 2. With a confidence level of 95%, an estimated proportion of 50%, and a 5% level of precision. The minimum sample size was calculated to be 385. However, more participants and candidates were included to ensure the sufficiency and accuracy of the results. Since it was an online survey, where the link was shared, it would be difficult to estimate the response rate.

Inclusion and exclusion criteria

Any patient with a previous diagnosis of sickle cell disease, consenting to participate in the study was included. Carriers of sickle cell trait and those free from trait and disease were excluded. Participants having sickle cell disease with any of the following diseases (thalassemia, chronic venous insufficiency, and systemic lupus erythematosus) were not included in the analysis for the part screening for leg ulcers and its associated history as they might be the cause of the ulceration and not sickle cell disease.

Statistical analysis

Data analysis was performed using Statistical Package for the Social Sciences (SPSS) version 23 (IBM Corp., Armonk, NY). Frequency and percentages were used to display categorical variables. Chi-square test was used to test for the presence of an association between having leg ulcers and sociodemographic variables as well as SCD-related history. Furthermore, binary logistic regression was utilized to determine predictive factors predicting the incidence of leg ulcers among SCD, the following variables were entered to the model (gender, age, being repetitively hospitalized due to SCD, frequency of hospitalization per year, frequency of vaso-occlusive per month, history of acute chest syndrome, history of priapism, history of stroke, utilization of hydroxyurea). Model fitness-of-good was tested using Omnibus test and Hosmer and Lemeshow test. The level of significance was set at 0.05.

## Results

A total of 3245 participated in the study, of which 790 had sickle cell disease, fitted the inclusion criteria, and therefore were included in the study. 505 were excluded for being carriers and 1935 were excluded as they were free from both the trait and the disease. Table 1 shows the sociodemographic profile of the participants having sickle cell disease. 261 (33%) were males and 529 (67%) were females. The age distribution of participants was as following 85 (10.8%) were 18 years old and younger, 236 (29.9%) were between 19 and 24 years, 132 (16.7%) were between 25 and 30 years, 200 (25.3%) were between 31 and 40 years, 107 (13.5%) were between 41 and 50 years and 30 (3.8%) were older than 50 years. 695 (88%) were Saudi and 95 (12%) were not Saudi. As for the place of residency, 693 (87.7%) were from the Eastern Region, 37 (4.7%) were from the Western Region, 33 (4.2%) were from the Northern Region and 27 (3.4%) were from the Southern Region. 383 (48.5%) were single, 390 (49.4%) were married and 17 (2.2%) were divorced or widowed. 102 (12.9%) had primary - intermediate school education, 225 (28.5%) had high school education, 438 (55.4%) had diploma / bachelor’s degree and 25 (3.2%) had master/Ph.D. degree.

**Table 1:** Sociodemographic profile of the participants having sickle cell disease (n = 790)

Demographical Characteristics		n	%
Gender			
Male		261	33
Female		529	67
Age			
18 years and younger		85	10.80
19 – 24		236	29.90
25 – 30		132	16.70
31 – 40		200	25.30
41 – 50		107	13.50
Older than 50 years		30	3.80
Nationality			
Saudi		695	88
Non-Saudi		95	12
Place of Residency			
Eastern Region		693	87.70
Western Region		37	4.70
Northern Region		33	4.20
Southern Region		27	3.40
Marital Status			
Single		383	48.5
Married		390	49.4
Divorced / Widowed		17	2.2
Education			
Primary - Intermediate School		102	12.90
High School		225	28.50
Diploma / Bachelor’s degree		438	55.40
Master / Ph.D. Degree		25	3.20

Table 2 shows the hospitalization and vaso-occlusive crisis history among sickle cell disease patients. When asked if the participants do repetitively get hospitalized due to SCD, 566 (71.6%) answered yes. The average frequency of hospitalization due to SCD is as following 236 (29.9%) answered 0 times, 358 (45.3%) answered 1-2 times, 114 (14.4%) answered 3-5 times, 40 (5.1%) answered 6-8 times and 42 (5.3%) answered more than 8 times. The average frequency of painful vaso-occlusive crisis due to SCD is as following 227 (28.7%) answered 0 times, 405 (51.3%) answered 1-2 times, 98 (12.4%) answered 3-5 times, 37 (4.7%) answered 6-8 times and 23 (2.9%) answered more than 8 times.

**Table 2:** Hospitalization and vaso-occlusive crisis among sickle cell disease (n = 790)

Question	n	%
Q1/ Do you get repetitively hospitalized due to SCD?		
Yes	566	71.6
No	224	28.4
Q2/ On average how frequently do you get hospitalized per year?		
0	236	29.9
1 - 2 times	358	45.3
3 - 5 times	114	14.4
6 - 8 times	40	5.1
More than 8 times	42	5.3
Q3/ On average how frequently do you get pain crises per month?		
0	227	28.70
1 - 2 times	405	51.30
3 - 5 times	98	12.40
6 - 8 times	37	4.70
More than 8 times	23	2.9

**Figure 1:**
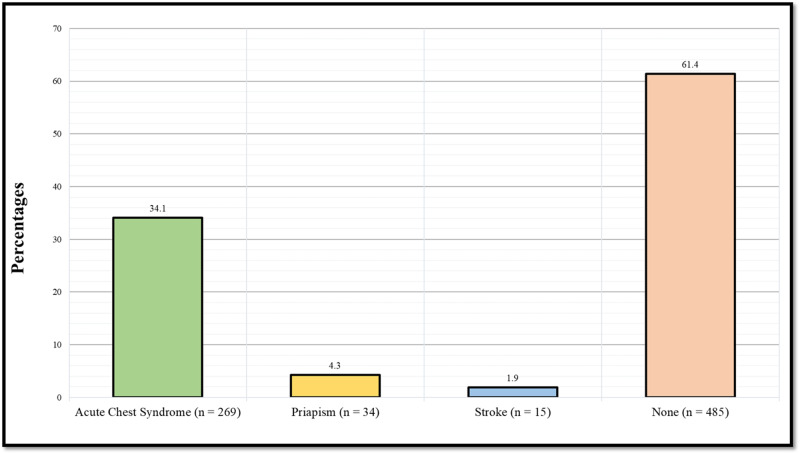
Prevalence of sickle cell disease complications among sickle cell disease patients

Figure 1 illustrates the prevalence of sickle cell disease complications among diseased patients. 269 (34.1%) have previously suffered from acute chest syndrome, 34 (4.3%) have previously suffered from priapism, 15 (1.9%) have previously suffered from a stroke, whilst 485 (61.4%) have never suffered from any of the mentioned complications.

**Figure 2:**
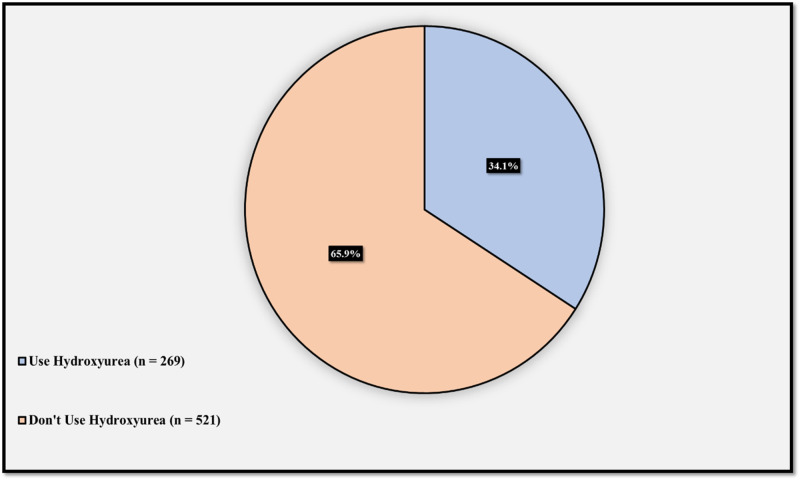
Utilization of hydroxyurea among sickle cell disease patients

Figure 2 illustrates the utilization level of hydroxyurea among sickle cell patients. 269 (34.1%) uses hydroxyurea, whilst 521 (65.9%) don’t.

**Figure 3:**
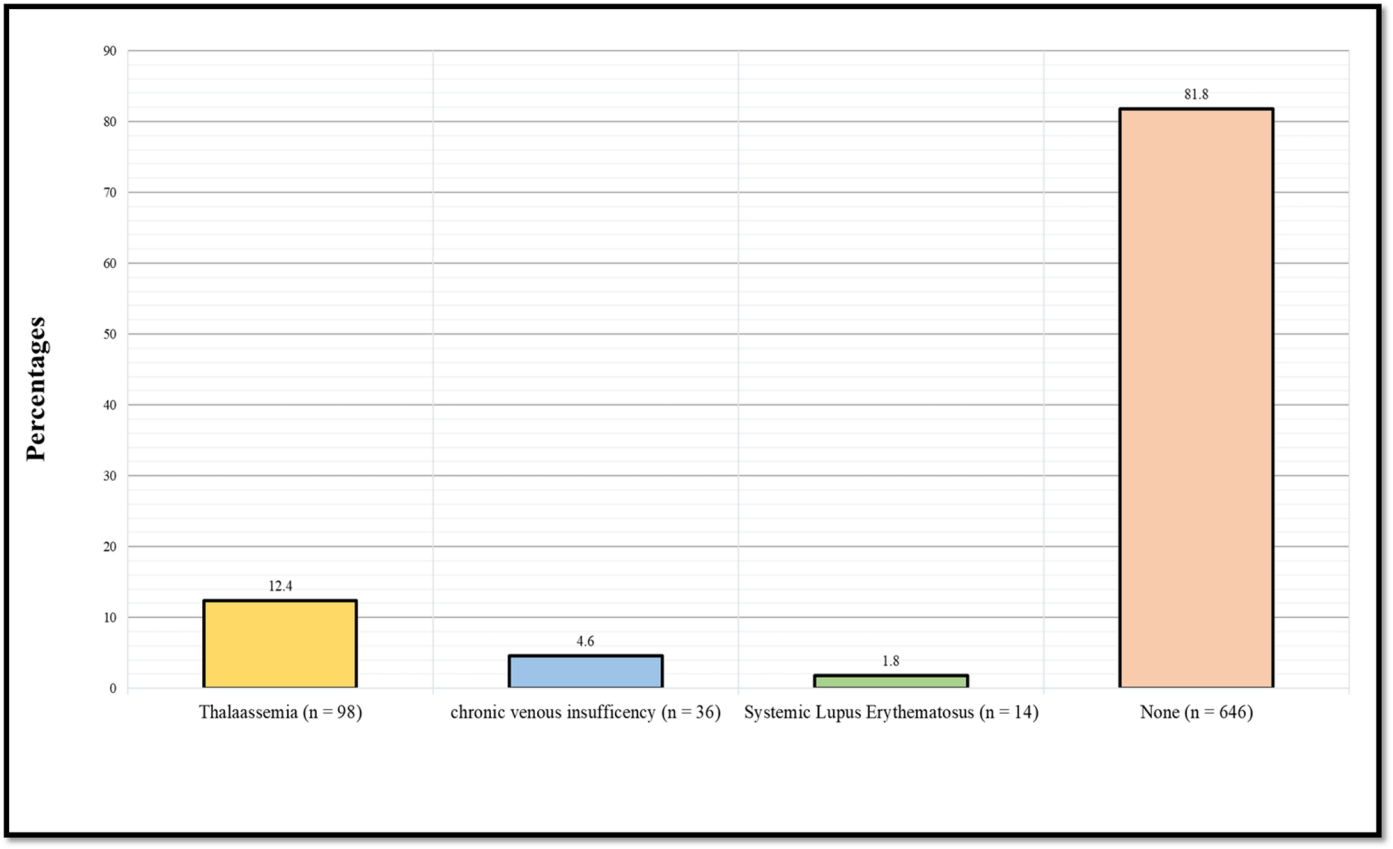
Prevalence of other diseases that can cause leg ulcers among sickle cell disease patients

Figure 3 demonstrates the prevalence of diseases that can cause leg ulcers among sickle cell disease patients other than sickle cell disease (thalassemia, chronic venous insufficiency, and systemic lupus erythematosus). 98 (12.4%) had thalassemia, 36 (4.6%) had chronic venous insufficiency, 14 (1.8%) had systemic lupus erythematosus, whilst 646 (81.8%) did not have any of the mentioned diseases. Those having any disease that can cause leg ulcers were excluded from further analysis (analysis of leg ulcer prevalence and associated history).

**Figure 4:**
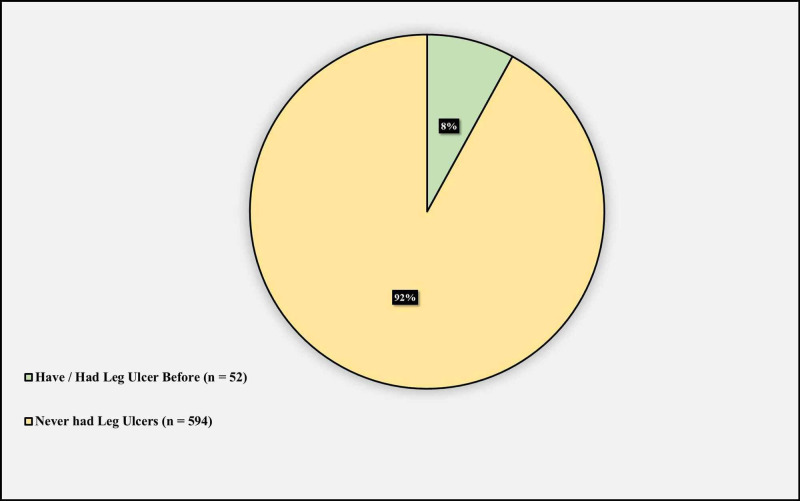
Lifetime prevalence of leg ulcers among sickle cell disease patients

Figure 4 demonstrates the lifetime prevalence of leg ulcers among sickle cell disease patients with no secondary causes of leg ulcers. 52 (8%) had leg ulcers, whilst 594 (92%) do not.

Table 3 displays the leg ulcer related history in SCD patients affected with leg ulcers. 33 (63.5%) suffered from leg ulcers 1-2 times. 8 (15.4%) suffered from leg ulcers 3-4 times and 11 (21.2%) suffered from leg ulcers more than 4 times. 35 (67.3%) stated the ulcer was painful and 17 (32.7%) stated it was not. Only 17 (32.7%) stated that there was an association between the incidence of leg ulcers and vaso-occlusive crisis. When asked which investigation was conducted in order to diagnose the leg ulcer, 6 (11.5%) said a biopsy was performed to be diagnosed, 5 (9.6%) said they did an ultrasound imaging to be diagnosed, whilst 41 (78.8%) were diagnosed clinically without investigation. When asked how the leg ulcer was treated, 11 (21.2%) reported analgesics as the mainstay of treatment, 37 (71.2%) reported topical treatment (creams and ointment) as the primary form of treatment, 3 (5.8%) reported having blood transfusion for the treatment of ulcer and 1 (1.9%) reported only dressing as the mainstay of treatment. As for the reported healing time, 10 (19.2%) said it was few days, 15 (28.8%) said it was one week, 6 (11.5%) said it was two to three weeks, 6 (11.5%) said it was one month, 6 (11.5%) said it was two to three months and 9 (17.3%) said it was more than three months.

**Table 3:** Leg ulcers history among sickle cell disease patients with leg ulcers (n = 52)

Question	n	%
Q1/ How many times have you suffered from leg ulcers?		
1 - 2 times	33	63.5
3 - 4 times	8	15.4
More than 4 times	11	21.2
Q2/ Was the ulcer painful?		
Yes	35	67.3
No	17	32.7
Q3/ Was the ulcer associated with VOC?		
Yes	17	32.7
No	35	67.3
Q4/ Which of the following have been conducted to diagnose you with leg ulcers? (more than one answer can be chosen)		
Biopsy	6	11.5
Ultrasound	5	9.6
Clinical Examination	41	78.8
Q5/ How was the ulcer treated?		
Analgesics	11	21.2
Topical Treatment (Creams and ointments)	37	71.2
Blood Transfusion	3	5.8
Dressing	1	1.9
Q6/ How long did the ulcer take to heal?		
Few days	10	19.2
1 week	15	28.8
2 - 3 weeks	6	11.5
1 month	6	11.5
2 - 3 months	6	11.5
More than 3 months	9	17.3

Table 4 displays the factors associated with the incidence of leg ulcers in patients with sickle cell disease. None of the sociodemographic variables were associated with the incidence of leg ulcers in sickle cell disease. As for the association between sickle cell disease-related history, only the average frequency of painful vaso-occlusive crisis was significantly associated with the incidence of leg ulcers (p < 0.001*). Using hydroxyurea was near significant (p = 0.056)

**Table 4:** Factors associated with the incidence of leg ulcers in patients with sickle cell disease (n = 646) *Significant at level 0.05

Demographical Characteristics		Have you ever suffered from leg ulcers?		P-value
		Yes	No	
Gender				0.268
Male		21 (9.7%)	195 (90.3%)	
Female		31 (7.2%)	399 (92.8%)	
Age				0.289
18 years and younger		5 (7.2%)	64 (92.8%)	
19 - 24		10 (5.1%)	186 (94.9%)	
25 - 30		11 (10.2%)	97 (89.8%)	
31 - 40		13 (7.9%)	151 (92.1%)	
41 - 50		9 (10.6%)	76 (89.4%)	
Older than 50 years		4 (16.7%)	20 (83.3%)	
Nationality				0.640
Saudi		47 (8.2%)	524 (91.8%)	
Non-Saudi		5 (6.7%)	70 (93.3%)	
Place of Residency				0.856
Eastern Region		47 (8.1%)	530 (91.9%)	
Western Region		3 (10.3%)	26 (89.7%)	
Northern Region		1 (4.2%)	23 (95.8%)	
Southern Region		1 (6.3%)	15 (93.8%)	
Marital Status				0.799
Single		25 (7.8%)	294 (92.2%)	
Married		27 (8.6%)	286 (91.4%)	
Divorced / Widowed		0 (0%)	14 (100%)	
Education				0.500
Primary - Intermediate School		6 (7.7%)	72 (92.3%)	
High School		18 (9.5%)	172 (90.5%)	
Diploma / bachelor’s degree		26 (7.2%)	333 (92.8%)	
Master / Ph.D. Degree		2 (10.5%)	17 (89.5%)	
Do you get repetitively hospitalized due to SCD?				0.148
Yes		42 (9%)	10 (91%)	
No		424 (5.6%)	170 (94.4%)	
On average how frequently do you get hospitalized per year?				0.079
0		8 (3.9%)	196 (96.1%)	
1 - 2 times		27 (9.3%)	263 (90.7%)	
3 - 5 times		11 (12.4%)	78 (87.6%)	
6 - 8 times		4 (12.1%)	29 (87.9%)	
More than 8 times		2 (6.7%)	28 (93.3%)	
On average how frequently do you get pain crises per month?				< 0.001*
0		8 (4.1%)	188 (95.9%)	
1 - 2 times		28 (8.4%)	307 (91.6%)	
3 - 5 times		4 (5.3%)	72 (94.7%)	
6 - 8 times		8 (28.6%)	20 (71.4%)	
More than 8 times		4 (36.4%)	7 (63.6%)	
Have you ever suffered from acute chest syndrome?				0.339
Yes		20 (9.5%)	190 (90.5%)	
No		32 (7.3%)	404 (92.7%)	
Have you ever suffered from priapism?				0.136
Yes		4 (16%)	21 (84%)	
No		48 (7.7%)	573 (92.3%)	
Have you ever suffered from a stroke?				0.642
Yes		1 (12.5%)	7 (87.5%)	
No		51 (8%)	587 (92%)	
Do you use hydroxyurea?				0.056
Yes		23 (11%)	186 (89%)	
No		29 (6.6%)	408 (93.4%)	

Table 5 displays the multivariate logistic regression for the prediction of leg ulcer incidence among sickle cell disease patients. The predictability of incidence of leg ulcers among SCD patients was tested using the following variables: gender, age, frequency of hospitalization per year, frequency of vaso-occlusive per month, history of acute chest syndrome, history of priapism, history of stroke, utilization of hydroxyurea was tested. The presence of the following characteristics was significantly more predisposing to leg ulcers: having painful vaso-occlusive crises 6-8 times per month or more than 8 times.

**Table 5:** Multivariate logistic regression (factors predicting incidence leg ulcers) *Significant at level 0.05

Variables				
Demographics	P-Value	Odds Ratio	Confidence Interval	
Gender (Male vs Female)	0.92	1.035	0.53	2.023
Age (18 Years and younger is the Referent)				
19 - 24	0.456	0.642	0.201	2.057
25 - 30	0.589	1.376	0.433	4.376
31 - 40	0.755	1.196	0.389	3.676
41 - 50	0.59	1.4	0.412	4.754
Older than 50 years	0.153	2.96	0.667	13.129
Sickle Cell Disease History	P-Value	Odds Ratio	Confidence Interval	
On average how frequently do you get hospitalized per year? (0 is the Referent)				
1 - 2 times	0.149	1.931	0.789	4.722
3 - 5 times	0.136	2.281	0.771	6.749
6 - 8 times	0.208	2.492	0.601	10.34
More than 8 times	0.345	0.395	0.057	2.718
On average how frequently do you get pain crises per month? (0 is the Referent)				
1 - 2 times	0.161	1.839	0.785	4.31
3 - 5 times	0.911	0.928	0.25	3.444
6 - 8 times	0.001*	8.206	2.499	26.952
More than 8 times	0.001*	19.218	3.321	111.226
Have you ever suffered from acute chest syndrome? (Yes vs No)	0.719	0.889	0.469	1.686
Have you ever suffered from priapism? (Yes vs No)	0.365	0.551	0.152	2.001
Have you ever suffered from a stroke? (Yes vs No)	0.844	1.291	0.102	16.419
Do you use hydroxyurea? (Yes vs No)	0.352	0.737	0.388	1.401

## Discussion

In this study, the lifetime prevalence of leg ulcers among SCD patients in Saudi Arabia was 8%. To the best of our knowledge, no previous studies have described the prevalence of leg ulcers in Saudi Arabia. However, other studies reported that the prevalence of leg ulcers among SCD patients in other countries varied and ranged from low to high. Previous studies revealed that leg ulceration among sickle cell patients prevalence was 2.5% in the United States [10], 3.5% in Italy, [11] 5.9% in Nigeria [12], 10.6% in Ghana [13], 13.2% in Sierra Leone [13], 29.5 % in Jamaica [14], and up to 43% in Brazil [15]. These observations further confirm the presence of a geographical variation with respect to clinical features of SCD, recognized by Nolan et al., Trent et al. and Ballas [8,16,17]. SCD genotype has been suggested by Minniti et al. as an explanation of this geographical variation, where patients within countries having specific SS genotype prevalence are more likely to be affected [18]. We too agree that the prevalence of specific genotypes according to the geographical region might have a bearing on leg ulcers, as with other phenotypic association with different genotypes of SCD. Another factor to put into consideration is the method of data collection, each study had its own way of data collection which might have contributed to some degree to this varying level of leg ulcer prevalence.

 In this study, although the prevalence of leg ulcers was higher in males, the gender variation was not statistically significant. This is in contrast with other studies where males were significantly more affected than females [10,12]. However, it is consistent with the finding of Halabi-Tawil et al. and Morgan [19,20]. Halabi-Tawil et al. mentioned in their study that this male predominance of leg ulcer prevalence is noted in studies conducted in North America but not in studies conducted in African countries and Jamaica. This makes the gender pattern seen in Saudi Arabia similar to the one seen in Africa and Jamaica.

Cumming et al. and Antwi-Boasiako stated in their study that SCD patients older than 18 were found to be at more risk for leg ulcers compared to younger patients. Thus, they suggest a correlation between age and leg ulcers [11,14]. These findings are not consistent with our study in which there was no significant difference in leg ulcer prevalence between different age groups, although a relative increase of prevalence was seen in older age groups.

In this study, we found no association between socioeconomic status (represented by income) and the prevalence of leg ulcers. This is in contrast with the findings of Minniti and Kato [21], Cumming et al. [14], and Olatunya et al. [12], where an association has been found between leg ulcers and socioeconomic status. Poor socioeconomic status has been identified as a risk factor for leg ulcers in their work; they explain this association through the assumption that people with low socioeconomic status have generally lack of care and poor nutritional intake. It is possible that the relatively high socio-economic status of the country in general and the subsidized health care system could be partly responsible for this finding in our study.

A significant association between painful vaso-occlusive crisis and prevalence of leg ulcers was observed in this study, where it has been found that a high rate of vaso-occlusive crisis per month is an independent risk factor for leg ulcers (particularly in those with six to eight times a month and more than 8 times a month). This finding opposes the findings of previous studies where an inverse relationship was found between vaso-occlusive crisis and leg ulcers according to Antwi-Boasiako et al, Olatunya et al, and Madu et al. [11,12,22]. They observed a significant decrease in the incidence of vaso-occlusive crisis in patients with leg ulcers. Antwi-Boasiako et al. explained this phenomenon by suggesting that vaso-occlusive crises in sickle cell disease are more commonly associated with a different phenotype as compared to leg ulcers and that vaso-occlusive crisis are related to viscosity while leg ulcers are related to hemolysis. This is one aspect that needs to be studied more and large genetic association studies would probably help to unravel the correlation between genotypes and phenotypes -in the context of specific clinical symptoms in SCD patients.

In our study, although those with SCD complications such as acute chest syndrome, stroke, and priapism had a relatively higher rate of leg ulcers, no significant correlation between leg ulcers and incidence of complications was present. However, previous studies have found a strong correlation between priapism, acute chest syndrome, and stroke with leg ulcers [23-25].

A relatively higher rate of leg ulcer incidence was observed in people using hydroxyurea, but this was not statistically significant. This is similar to the finding of Prabhash and Bapsy as well as the finding of Hwang et al., where they found that using hydroxyurea is associated with the incidence of leg ulcers, however, it is questionable whether using hydroxyurea in itself is contributing to the development of leg ulcers or if this association has another explanation as the association does not necessarily mean causation [26,27].

Limitations

The study has a self-reported cross-sectional design, which might subject the accuracy of the obtained data into question. Also, neither physical examination nor laboratory investigations were obtained and analyzed to confirm the diagnosis and determine the biomarkers highly associated with leg ulcers.

However, this study provides a good baseline about the prevalence of leg ulcers and we would recommend performing larger hospital-based studies where proper history taking, physical examination, and lab investigations are performed to identify the findings correlated with leg ulceration in SCD patients.

## Conclusions

Leg ulcers among SCD patients in Saudi Arabia were considerably prevalent (8%). There was no statistically significant correlation between leg ulceration and sociodemographic variables. Leg ulcers were more likely in patients with a history of highly frequent vaso-occlusive crises. No association was found between the incidence of leg ulcers and other complications of sickle cell disease or hydroxyurea.
